# 环状RNA与肺癌的研究进展

**DOI:** 10.3779/j.issn.1009-3419.2018.07.07

**Published:** 2018-07-20

**Authors:** 银玉 穆, 服役 谢

**Affiliations:** 315040 宁波，宁波市医疗中心李惠利医院检验科 Department of Clinical Laboratory, Ningbo Medical Center, Lihuili Hospital, Ningbo 315040, China

**Keywords:** 环状RNA, 肺肿瘤, 基因表达, 调控, Circular RNA, Lung neoplasms, Gene expression, Regulation

## Abstract

肺癌是目前世界上对人类健康和生命威胁最主要的恶性肿瘤之一，也是全世界目前因恶性肿瘤导致死亡的第一病因。目前认为其发生由很多因素共同作用的结果。环状RNA（circular RNA, circRNA）作为非编码RNA家族成员，具有特征性的共价闭合环结构，在各种生物中广泛表达。circRNA由于特殊的结构，具有高度的特异性、保守性和稳定性，可能在肺癌的发生发展过程中，具有潜在的临床价值。circRNA的主要功能是作为海绵吸附miRNA发挥转录调控作用，同时还具有调控可变剪接、编码蛋白等作用。目前circRNA在肺癌中的作用及机制并不是很清楚，本文就circRNA的特征、功能、作用机制以及在肺癌发生、发展中的作用进行综述。

## Abstract (en)

Lung cancer is one of the most important malignant tumors of human health and life in the world, and is the leading cause of death in the world. At present, it is believed that it is caused by many factors. Circular RNA (circRNA), as a class of non-coding RNA family, has covalently closed loop structure. CircRNA is abundant in different cells. CircRNAs due to the special structure, with a high degree of specificity, conservation and stability, may have a potential clinical value in the development of lung cancer. The biological function of circRNA is multi-faceted, including miRNA sponge, transcriptional and alternative splicing, protein coding, and so on. Currently, the role and mechanism of circRNA in lung cancer is not very clear. In this paper, the characteristics, function, mechanism and the role of circRNAs in the occurrence and development of lung cancer are reviewed.

环状RNA（circular RNA, circRNA）是一类不具有5' 末端帽子和3' 末端poly（A）尾巴并以共价键形成环形结构的RNA，广泛存在于真核细胞中。circRNA属于非编码RNA，部分非编码RNA参与基因转录后的调控、剪切和修饰等，在生命活动中发挥重要的作用。近几年来，研究的热点主要集中在微小核糖核酸（microRNA, miRNA）及长链非编码RNA（long noncoding RNA, lncRNA），它们广泛参与细胞分化、发育、衰老和肿瘤发生、发展等生命过程^[1-3]^。目前circRNA已成为RNA研究领域的新热点^[4-8]^，由于其具有特殊的结构，其在肿瘤诊断、治疗及预后中的潜在临床价值已受到密切关注^[9-14]^。

## 1 circRNA的来源及特征

20世纪70年代科学家在RNA病毒中发现了一种环形RNA分子，命名为circRNA^[15]^。其后，分别在酵母细胞和人体细胞中发现了circRNA^[16, 17]^。后续研究证实，circRNA在细胞中广泛存在。circRNA与传统线性RNA不同，其分子结构呈封闭环状，不含5′和3′末端，十分稳定，不易被核酸外切酶（RNase R）所降解^[18]^。随着分子生物学技术的发展，科学家发现了大量无尾巴的circRNA，并逐步揭示它们成环机制和在基因转录调控中的重要功能^[19-21]^。circRNA作为一类新型的RNA分子，具有高度的特异性、保守性和稳定性。大部分的circRNA是由外显子序列构成，在不同的物种中具有保守性，同时存在组织及不同发育阶段的表达特异性。由于circRNA对核酸酶不敏感，因此比线性RNA分子更为稳定，易出膜进入体液，使得circRNA在作为新型临床诊断标记物的开发应用上具有明显优势。

## 2 circRNA的功能

根据RNA的构成，circRNA主要分为3类：外显子环化RNA（exonic circRNA, circRNA）、内含子环化RNA（intronic circRNA, ciRNAs）和外显子-内含子环化RNA（exon-introncircRNA, EIciRNAs）。外显子环化RNA是由外显子转录的RNA构成，是最常见的circRNA。越来越多的研究表明，circRNA几乎参与整个生物过程，它们在转录、翻译及反转录等过程中都有潜在的调控作用。

### 2.1 circRNA作为内源性竞争RNA（endogenous competitive RNA, ceRNA）影响miRNA转录后调控功能

circRNA是一类长度21 nt-25 nt的小非编码RNA，与mRNA进行碱基互补配对，抑制其翻译，还可引起靶mRNA降解。有些circRNA具有miRNA应答元件，能够结合（吸附）游离miRNA，从而阻断miRNA对基因表达的抑制。如小脑变性相关蛋白反义转录物（CDR1as）可作为miR-7海绵，即ciRS-7。ciRS-7可抑制miR-7活性并与miR-7竞争结合其他RNA，从而调控靶基因的表达^[22]^。此外，田芳等^[23]^报道，环状RNA CircHIPK3等也具有海绵吸附作用，能够结合miR-379，调控IGF1表达促进细胞增殖。circRNA上面有多个miRNA结合位点，而且circRNA对miRNA吸附能力比其他的ceRNAs强大，所以也被称为"超级海绵"。

### 2.2 circRNA可与RNA结合蛋白结合形成RNA蛋白复合体进而影响基因的表达水平

这种复合体能够调控RNA以及RNA结合蛋白之间的相互作用，从而影响转录后的基因表达水平^[24]^。

### 2.3 circRNA内部包含核糖体进入位点可翻译表达蛋白质

天然的circRNA不能和核糖体结合，也不能翻译蛋白质，但具有翻译的起始密码子，人工合成的含有核糖进入位点（IRES）的circRNA在体外实验中能够翻译蛋白质^[25]^。最新的研究也证实了circRNA在真核细胞中具有编码蛋白质功能。Legnini等^[26]^发现，circ-ZNF609具有一个开放性的阅读框（open reading frame, ORF），两端分别含有终止密码子和起始密码子，通过多聚核糖体翻译合成蛋白质。另外，Yang等^[27]^还发现，N6-甲基腺嘌呤促进circRNA翻译的启动。

### 2.4 circRNA参与调控选择性拼接与基因转录

ciRNAs和EIciRNAs主要位于细胞核内，目前已被证实能够调控亲本基因的转录。Qu等^[28]^报道，EIciRNAs中的circPAIP2和circEIF3J能够与U1snRNP结合形成复合物，此复合物与RNA聚合酶Ⅱ相互作用，促进亲本基因的转录。在前体RNA剪接过程中，反向剪接产生的circRNA能够竞争性调控可变剪接。Conn等^[29]^研究发现，来源于SEP3外显子6的circRNA可以结合其同源的DNA形成一个RNA:DNA杂交物或R环。R回路的形成可导致转录暂停，这些circRNAs可能有助于提高其同源外显子的拼接效率，从而竞争性调控可变剪接。

### 2.5 其他功能

有些circRNAs参与运输miRNA，CDR1as作为分子海绵吸附大量miRNA-7，CDR1as被miRNA-671降解，而释放大量的miRNA-7，相当于起到miRNA-7转运载体的作用^[30]^。

## 3 circRNA的筛选与验证

由于没有3′末端，传统RNA测序技术无法检测出circRNA。研究人员通过分析RNA测序数据，在测序结果中寻找两个重排外显子之间的结点，来识别可能存在的circRNA^[31-35]^。目前有许多商业化筛选手段，如高通量测序、基因芯片。高通量测序成本较高，但可以发现新的circRNA，芯片成本相对较低，但只能检测已知的circRNA。筛选出的circRNA可以通过荧光定量PCR技术进行验证。虽然circRNA筛选技术有所发展，但现阶段研究circRNA仍然存在一些问题，如标本中circRNA表达量较低，不便于检测; 母源基因表达和功能之间的关系还不明确等。

## 4 circRNA与肺癌的研究

非编码RNA在肿瘤发生发展中的作用及机制是目前分子生物学领域的研究热点之一，关于miRNA及lncRNA与肿瘤的相关性研究已取得大量成果。而circRNA在肿瘤中的研究也成为RNA领域新的焦点。目前关于circRNA在肺癌中的研究也还在起步阶段。

Zhao等^[36]^通过高通量测序，研究早期肺腺癌组织中的circRNA表达谱，通过生物信息学，分析筛选出差异表达的circRNA。他们发现有357个circRNAs表达失调，并挑出5个特异性circRNAs进行生物学分析，并预测了circRNA-miRNA具有潜在的相互作用，这些circRNAs可能为肺癌的早期诊断提供潜在的靶点。

基于circRNA对转录后调控作用，陈丽剑等^[35]^研究发现，在苯并[a]芘致肺癌中circRNA 001988的表达下调，提示该基因可能具有抑癌基因的作用。他们又进一步使用生物学信息软件RegRNA预测得到这些microRNA下游的与癌症的发生密切相关的150多个mRNA，他们选择了与癌症相关性较高的一个mRNA（CCDC6）做了进一步的检测，证明它对细胞周期具有重要的调控作用。hsa-miR-382-5p和hsa-miR-583在癌细胞中的表达上调，可能是由于circRNA 001988下调之后，对其靶向的miRNA的吸附作用减弱，使得miRNA表达上调。这些结果均表明，circRNA 001988可通过调节miRNA活性，从而发挥抑癌作用。

另外，Wan等^[37]^研究发现，cir-ITCH在肺癌组织中的表达明显下降。cir-ITCH通过Wnt/Catenin通路抑制肺癌增殖。他们通过细胞研究证明cir-ITCH也在不同肺癌细胞株中表达增强。cir-ITCH的异位表达，明显提高了抑制癌基因的表达，从而抑制癌细胞的增殖。他们从分子水平研究显示，cir-ITCH作为miR-7和miR-214海绵，提高*ITCH*基因的表达，从而抑制Wnt/β-catenin信号通路的激活。Yao等^[38]^报道，在非小细胞肺癌组织中circRNA100876表达显著上调，并与肿瘤分期明显相关。这些进一步表明circRNA在肺癌发生、发展过程、诊断及治疗中具有重大意义。

此外，Zhu等^[39]^报道，肺腺癌组织中hsa_circ_0013958明显上调，并与TNM分期明显相关。所以在肺腺癌的不同阶段，相关circRNA表达量也可能会有所不同。circRNA表达量的不同可能对肺腺癌的严重程度起预测作用。因此，肺腺癌患者外周血中的差异表达的circRNAs可能成为诊断肺腺癌的潜在生物标志物。

## 5 展望

由于circRNA具有多样性，我们推测还会有更多的circRNA参与肺癌的发生和发展。目前circRNA在肺癌中的研究才刚刚起步。利用新发展的高通量测序，可以筛选出新的可能与肺癌相关的circRNA。在细胞或动物实验中，通过调控其转录水平，深入了解其在肺癌发生和发展中起到的作用及机制，对于肺癌的诊断、治疗可能具有重要的临床价值。
